# Visual perception of travel distance for self-motion through crowds

**DOI:** 10.1167/jov.23.4.7

**Published:** 2023-04-26

**Authors:** Anna-Gesina Hülemeier, Markus Lappe

**Affiliations:** 1 Department of Psychology, University of Münster, Münster, North-Rhine Westphalia, Germany; 2 Department of Psychology, University of Münster, Münster, North-Rhine Westphalia, Germany

**Keywords:** optic flow, distance perception, biological motion, point-light walkers, leaky path integration

## Abstract

Humans can use visual motion to estimate the distance they have traveled. In static environments, optic flow generated by self-motion provides a pattern of expanding motion that is used for the estimation of travel distance. When the environment is populated by other people, their biological motion destroys the one-to-on correspondence between optic flow and travel distance. We investigated how observers estimate travel distance in a crowded environment. In three conditions, we simulated self-motion through a crowd of standing, approaching, or leading point-light walkers. For a standing crowd, optic flow is a veridical signal for distance perception. For an approaching crowd, the visual motion is the sum of the self-motion–induced optic flow and the optic flow produced by the approaching walkers. If only optic flow were to be used, travel distance estimates would be too high because of the approaching direction of the crowd toward the observer. If, on the other hand, cues from biological motion could be used to estimate the speed of the crowd, then the excessive optic from the approaching crowd flow might be compensated. In the leading crowd condition, in which walkers of the crowd keep their distance from the observer as they walk along with the observer, no optic flow is produced. In this condition, travel distance estimation would have to rely solely on biological motion information. We found that distance estimation was quite similar across these three conditions. This suggests that biological motion information can be used (a) to compensate for excessive optic flow in the approaching crowd condition and (b) to generate distance information in the leading crowd condition.

## Introduction

Spatial navigation requires the monitoring of motion direction and travel distance. Sensory information to estimate travel distance during self-motion consists of vestibular cues (Israël & Berthoz, 1989; Harris, Jenkin, & Zikovitz, 2000; Frissen, Campos, Souman, & Ernst, 2011; Cheng & Gu, 2018), proprioceptive cues (Thomson, 1980; Frissen et al., 2011), self-generated (“idiothetic”) cues (Mittelstaedt & Mittelstaedt, 1973; Nico, Israël, & Berthoz, 2002), and visual cues (Bremmer & Lappe, 1999; Harris et al., 2000; Lappe, Jenkin, & Harris, 2007; Redlick, Jenkin, & Harris, 2001; Frenz, Bremmer, & Lappe, 2003). Perceiving self-motion from visual cues entails the analysis of optic flow (Gibson, 1950; Frenz & Lappe 2005), the pattern of expanding visual motion generated during forward locomotion.

Because, strictly speaking, the optic flow is ambiguous for estimating travel distance, it requires some way of scaling the visual environment (i.e., something like a yardstick) (Lee, 1974; Lee, 1976; Lee, 1980). This scaling is available, for example, from one's eye height if the environment contains a ground plane (Frenz & Lappe, 2005). In such a case, optic flow speed can be transformed into ego-speed.

Ego-speed obtained from optic flow must be integrated over space (Lappe, Lappe, Kolesnik, & Bührmann, 2007; Lappe, Stiels, Frenz, & Loomis, 2011) to provide traveled distance (Ellmore & McNaughton, 2004). Researchers refer to this integration process as “path integration” (Maurer & Séguinot, 1995; Bremmer & Lappe, 1999; Kearns, Warren, Duchon, & Tarr, 2002; Ellmore & McNaughton, 2004; Lappe et al., 2007). Path integration is a theoretical mechanism that might underpin the estimation of traveled distance during locomotion (Mittelstaedt & Mittelstaedt, 2001). Because travel distance discrimination requires estimating self-motion signals relative to the environment, path length estimation is not directly derived from the image motion but rather from the observer's self-motion (Frenz et al., 2003; Mossio, Vidal, & Berthoz, 2008, Lappe et al., 2011).

Although many experiments have shown that optic flow provides access to travel distance, these experiments have also revealed increasing underestimation of travel distance with increasing true travel distance. This underestimation has been observed in real environments (Lappe & Frenz, 2009), simulations (Frenz & Lappe, 2005; Lappe, Frenz, Bührmann, & Kolesnik, 2005), and virtual reality experiments (Redlick et al., 2001; Frenz & Lappe, 2006; Frenz et al., 2007; Lappe et al., 2007; Steinicke, Bruder, Hinrichs, Lappe, Ries, & Interrante, 2009). Lappe et al. (2007) proposed a leaky path integration model to explain travel distance estimates and their increasing misestimation over long travel distances.

According to the leaky integration model, two parameters influence the instantaneous change of distance: the gain factor (*k*) and the leak rate (α). The gain factor (*k*) describes the transformation from optic flow to ego-speed and, thus, how the integrated distance is incremented proportionally to the distance of the observer's motion. The leak rate (α) describes how the integrated distance reduces as the motion continues. Because of the leak, longer distances lead to a greater decrease in the current distance estimate such that the extent of underestimation increases. The leak accumulates over the path that is traversed (Lappe et al., 2011).

Empirical studies and computational models have examined traveled distance estimation from optic flow analysis in static environments. In natural environments, however, we are often confronted with dynamic scenes in which other people walk alongside us. The motion of other walking humans, known as biological motion (Johansson, 1973), introduces noise to the optic flow field, thereby biasing optic flow analysis for heading, the direction of one's self-motion (Riddell & Lappe, 2017; Riddell & Lappe, 2018; Riddell, Li, & Lappe, 2019; Hülemeier & Lappe, 2020; Koerfer & Lappe, 2020) and concerning flow parsing, the estimation of independent object motion within a flow field (Mayer, Riddell, & Lappe, 2021). Biological motion consists of limb articulation and its associated translation through space. In natural locomotion, articulation and translation are linked such that the articulation delivers cues about the speed and direction of the walker (Giese & Lappe, 2002; Masselink & Lappe, 2015; Thurman & Lu, 2016). Humans can derive the corresponding translation speed and direction from limb articulation alone (Fujimoto & Sato, 2006; Giese & Lappe 2002; Masselink & Lappe, 2015; Thurman & Lu, 2016). In the present study, we asked whether these biological motion cues can be used for travel distance estimation in combination with the optic flow. Because biological motion conveys information about the walking speed of a person that approaches or walks ahead of oneself, the speed information in the biological motion might also be integrated to derive travel distance. Consider a situation where you walk among a crowd of people coming toward you. Taking the optic flow perspective, an approaching crowd produces much optic flow as the walkers move toward you in addition to your self-motion. In contrast, if you follow a crowd of people walking in front of you at the same speed as yourself, the group does not cause any optic flow. Yet, the biological motion of the crowd contains cues to their walking speed and, by extension, to the travel distance. In the present experiments, we compared crowds of walkers that come toward the observer with crowds that walk in front of the observer and with static scenes in which the observer moves through a crowd of standing people.

## Methods

### Sample

We recruited 25 participants (eight males, 17 females) from the University of Münster. Ages ranged from 18 to 35 years (*M* = 21.28, *SD* = 4.16). All participants were naïve regarding the aim of the experiment. Their visual acuity was normal or corrected to-normal. Everyone gave written informed consent. Ethics approval was obtained from the ethics board of the Department of Psychology and Sport Science at the University of Münster. Participation was voluntary, anonymous, and compensated by either course credits or money.

### Setup

Experimental testing took place in a quiet, darkened room. We generated stimuli in MATLAB R2020a (The MathWorks, Natick, MA) with the Psychophysics Toolbox V3 (Kleiner, Brainard, & Pelli, 2007) and the OpenGL libraries (version 2.1) add-ons. Stimuli were projected onto a 250 cm × 200 cm backlit screen by a Marquee 8500 projector (VDC Display Systems, Tucker, GA) connected to a MacBook Pro (equipped with a 512-MB Intel HD graphic card; Apple, Cupertino, CA). The screen resolution was 800 × 600 pixels with a frame rate of 120 Hz. Participants sat 100 cm away from the screen on a chair resulting in a visual field of 102° by 90°. They registered their responses by moving the cursor up or down and pressing the left button of a computer mouse.

### Scene

**Figure 1.**
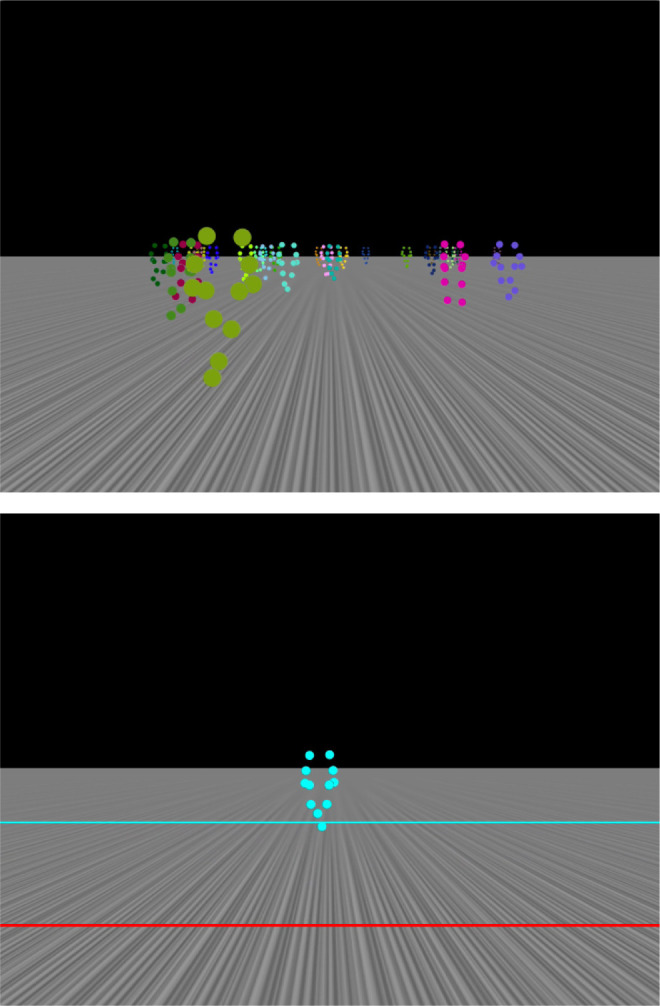
Stimulus and reporting procedure. The upper panel shows the crowd of point-light walkers placed on the ground plane. The stripes on the ground plane were oriented such that they provide perceptive depth information but no optic flow. The lower panel shows the procedure for reporting perceived travel distance. The red line represents the reference line from which the participants started. This red reference line appeared only during the distance estimation and not during motion simulations. Participants moved the blue line along with the walker to indicate their traveled distance. The size of the walker scaled with its depth.

The simulated virtual world spanned over 60-meter scene depths. We placed the horizon of a visible ground plane at eye height (1.60 meters). The ground structure consisted of stripes oriented in the motion direction, (Figure 1, Supplementary Movie S1) so that the ground provided static information about distance via perspective cues but no optic flow.

### Point-light walkers

Point-light walkers were constructed from the motion-tracking data of a single walking human (De Lussanet, Fadiga, Michels, Seitz, Kleiser, & Lappe, 2008). Each walker consisted of 12 points corresponding to the ankles of a human body (knee, hip, hands, elbow, and shoulder joints). We created a crowd of 30 walkers. To allow easier identification and segmentation of the walkers in the crowd, each walker had a unique color different from the others. Each walker started individually with a random selection starting position in the gait cycle. The group appeared collectively as static, approaching or leading the observer.

#### Walker speed

Walkers walked with natural speed but slight variations so that the group contained different paces. To implement this, the original motion-tracking data, which had a translation speed of 0.04 m/s, was interpolated with either 0.8 times (slower) or 1.2 times (faster) the original articulation and translation speed. The three speeds were then distributed equally across the 30 point-light walkers that formed the crowd. Thus, 10 walkers walked at the original speed, 10 at a slightly faster pace, and 10 at a slightly slower pace. The average speed of the crowd was always the original speed. Starting position in the gait cycle was randomized across the walkers.

#### Walker position

The walkers were uniformly distributed within the viewing frustum up to 26 meters in distance from the observer. This limited in-depth position is beneficial for leading crowds combined with slow observer speeds. When walkers disappeared from the frustum, we replaced them at 23 meters in depth from the observer. To avoid collisions with static or approaching walkers, we created a 3-meter-wide path to keep observer movements clear of walkers. Yet, shortly before the end of the trial (less than 10 seconds left), walkers disappearing from the frustum were allowed to be placed on the path as there was no more risk of collision. This setting did not reveal any information about travel distance or travel velocity, but it made the scene look more natural. Leading crowds did not need any replacements within the frustum.

### Self-motion simulation

We simulated the observer's forward self-motion at different speeds and duration to obtain a set of travel distances that could be reached with different combinations of speed and duration as in Lappe et al. (2007). Travel distances were 4.00, 5.66, 8.00, 11.31, 16.00, and 22.63 meters. Each distance was simulated with two different speeds and respective durations. Distances of 4.00, 5.66, and 8.00 meters were simulated with 0.8 m/s and 0.4 m/s. Distances 11.31, 16.00, and 22.63 meters were simulated with 0.8 m/s and 1.2 m/s. We chose these combinations to avoid very short and excessively long trial durations. Depending on travel distance and velocity, a trial lasted between 5 and 28 seconds.

### Conditions

We combined three walker conditions (approaching vs. leading vs. static) with six traveled distances (4.00, 5.66, 8.00, 11.31, 16.00, and 22.63 meters), and two self-motion speeds for each distance. This combination of variables resulted in 36 trials. We repeated each stimulus combination five times in random order giving 180 trials in total.

### Procedure

Participants saw a motion trial and afterward reported how much distance was covered. They indicated the perceived travel distance by placing a point-light walker along with a blue indicator line extending from the walker's feet such that its position in depth from a red starting line presented 2.624 meters in front of the observer matched the perceived distance covered in the travel simulation (“adjust-to-target” paradigm) (Lappe et al., 2007; Lappe & Frenz, 2009). The size of the walker scaled with its position in depth.

We instructed participants orally and in writing about the study procedure and their tasks. After the instruction, participants completed a practice block without data collection and performance feedback. The practice block contained 10 trials in randomized order. We presented the same practice trials to all participants so they all had the same anchor for distance estimation (see Table 1).

**Table 1.** Composition of the practice trials.

Walker condition	Travel distance (m)	Travel velocity (m/s)
Leading	5.66	0.8 (equal)
Static	8.00	0.8 (equal)
Leading	8.00	0.8 (equal)
Approaching	11.31	0.8 (equal)
Static	16.00	0.8 (equal)
Approaching	5.66	0.4 (slower)
Static	8.00	0.4 (slower)
Leading	8.00	0.4 (slower)
Leading	11.31	1.2 (faster)
Static	16.00	1.2 (faster)

Data collection started after the practice block. All stimulus combinations were repeated five times and distributed over two blocks (the first block with three repetitions of stimulus combinations and the second block with two repetitions). The whole session took about 1.75 hours, including short breaks between blocks. We compensated participants with either money or course credits.

### Leaky integration model

From previous studies (Lappe et al., 2007; Lappe et al., 2011; Harris et al., 2012; Bossard, Goulon, & Mestre, 2016; Bossard & Mestre, 2018), we expected that perceived traveled distance is not linearly related to true travel distance. Therefore, we fitted each participant's data to the leaky integration model and then computed traditional inferential analyses with the calculated parameters from the fit. According to the leaky integration model, perceived distance *p*(*x*) for a true distance *x* is

$$p\left(x\right)=e^{-\alpha x+b}+\frac{k}{\alpha }$$

The leaky integration model has two parameters: *k* and α. Gain factor *k* describes to what extent physical and perceived distances are congruent. Values around 1 indicate perfect congruency, values above 1 indicate distance overestimation, and values smaller than 1 denote distance underestimation. The leak parameter (α) measures the extent to which the perceived traveled distance is reduced throughout the movement. If α is greater than 0, the perceived traveled distance will become disproportionately smaller while moving. Parameter α is also an indicator of whether the relationship between perceived and true travel distance is linear. If it were, the best-fitting α would be zero.

### Fitting procedure

Analogously to Lappe et al. (2007), we collapsed data over velocity. For each participant and walker condition, we calculated a leaky model fit (as described by Lappe et al., 2007). Before proceeding with analyses, we tested α against zero to determine whether the data were either linear (α = 0) or nonlinear (α > 0). The Shapiro–Wilk normality test showed that the distribution of α departed significantly from normality (*W* = 0.90, *p* < 0.001). Results from the one-tailed Wilcoxon signed-rank test confirmed (*W* = 300, *p* < 0.001 across walker conditions) that α was significantly larger than zero, and our fit is, thus, nonlinear. This finding further supports our assumption that participants disproportionally misjudged their traveled distance with ongoing self-motion.

### Data preparation and check

We checked participant-wise the relation between estimated and traveled distance by calculating Kendall's τ as the correlation coefficient. The static walker condition served as the reference because it provided pure optic flow. Due to poor performance in the static condition (Kendall's correlation between traveled and estimated distance *r*_τ_ = 0.008; 95% confidence interval [CI], –0.09 to 0.25; *z* = 0.89; *p* = 0.371), we excluded one participant from further analyses. In 24 out of 9000 trials, distance estimate values were negative because the blue indicator lit with the walker was placed closer than the reference line. These cases probably occurred from pressing the mouse button by mistake and were removed from the analysis.

### Analysis procedure

The inferential analysis concentrated on the gain factor *k* and the leak rate α from the leaky integration fit. The data structure is based on a within-subject design with repeated measurements and two categorical independent variables with several levels. We performed an analysis of variance by applying a mixed-modeling framework (LMM). LMM benefits from higher flexibility, accuracy, and power for repeated-measures data (Kristensen & Hansen, 2004; Jaeger, 2008) than traditional variance analyses.

We analyzed whether the magnitude of *k* or α depends on the walker conditions. For this calculation, we fitted LMM (estimated using restricted maximum likelihood [REML] criterion and nloptwrap optimizer) with random intercept and constant slope for participants. Because of the data structure it was impossible to further cluster the observations by random effects. We incorporated walker conditions with three levels (static vs. approaching vs. leading) as factors. The static walker condition was set as reference. The model included participant ID as a random effect and conditions as fixed effects. In other words, we assumed that *k* and α have some residual variation associated with participants. By using participants as random effects, we modeled the unexplained variation of *k* and α through the variance of the participants. We obtained standardized parameters by fitting the model on a standardized version of the dataset. The 95% CIs and *p*-values were aligned to the Wald approximation. Effect sizes were labeled following Field's (2013) recommendations. Significant main effects were followed by post hoc analyses with the Tukey method for *p*-adjustments (two-tailed testing).

## Results

### Distance estimation in the presence of static walkers replicates previous findings of travel distance estimation from optic flow

In the static condition, walkers were standing in place while observer travel was simulated through the static crowd. In this condition, travel distance estimation is based only on optic flow. Therefore, we first checked whether distance estimates were consistent with previous studies of travel distance estimation from optic flow (Frenz & Lappe, 2005; Lappe et al., 2005; Lappe et al., 2007; Lappe & Frenz, 2009; Bossard et al., 2016).

**Figure 2.**
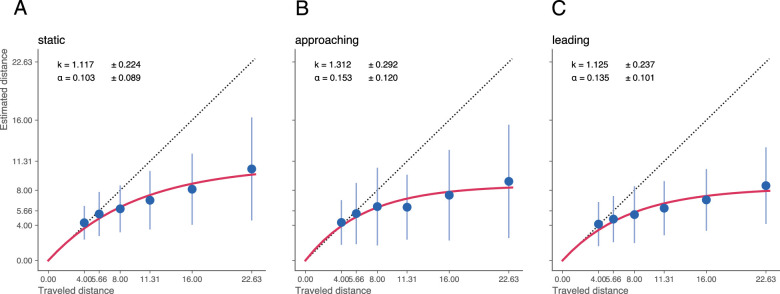
Descriptive results and model fits per condition. The points show the average distance estimates. The error bars give the standard deviation of the average distance estimates. The solid red line indicates the fit of the leaky integration model. The black dotted line represents veridical performance.

In those studies, perceived travel distance was progressively underestimated as true travel distance increased. This tendency was also observed in our data (Figure 2A). For the shortest distance (4 meters), participants on average slightly overestimated travel distance by 7.5% (0.3 meter). For longer distances, travel distance was more and more underestimated, reaching the largest underestimation (53.91% or 12.20 meters) for the longest travel distance (22.63 meters).

These data were fit by the leaky path integration model. The fit gave a gain factor of *k* = 1.117 (*SD* = 0.22) and a leak rate α above zero (*M* = 0.103, *SD* = 0.09). These values are close to those of previous optic flow-based studies that reported gain factors around 1 and leak rates between 0.02 and 0.22 (Lappe et al., 2007; Lappe et al., 2011; Bossard et al., 2016; Bossard & Mestre, 2018; Clément, Bukley, Loureiro, Lindblad, Sousa, & Zandvilet, 2020; Stangl, Kanitschneider, Riemer, Fiete, & Wolbert, 2020).

### In the approaching crowd condition, biological motion perception compensates for the increase in optic flow

When the observer traverses a crowd of walkers that approach them, the optic flow that the observer experiences is the sum of the optic flow produced by the observer's motion and the optic flow generated by the approaching walkers. If travel distance estimation used only optic flow, then the distance should be overestimated. This was not the case. Instead, distance estimates were below the veridical performance (Figure 2B). Thus, the biological motion of the walkers, particularly their articulation, must have been used to compensate for the excessive optic flow introduced by the crowd motion.

### In the leading crowd condition, biological motion perception allows the estimation of travel distance despite a lack of optic flow

When the observer follows a leading crowd of walkers that walks in front of them, the walkers in the crowd produce little or no optic flow as they keep their distance from the walker. If the observer bases their travel distance estimate only on optic flow travel distance estimation would not be possible. Yet, the reported travel distance in this condition (Figure 2C) was again quite similar to that of the static condition, in which optic flow was available as the only cue. Therefore, in the leading crowd condition participants must have used biological motion perception to estimate travel distance.

### Fit parameters show the interplay between optic flow and biological motion

In all conditions, distance estimates are well described by the leaky integration model. A one-tailed Wilcoxon signed-rank test (*W* = 300, *p* < 0.001; Shapiro-Wilk normality test indicated that the distribution of α departed significantly from normality: *W* = 0.90, *p* < 0.001) across walker conditions confirmed that α was significantly larger than zero, and our fit is, thus, nonlinear. This finding corroborates the observation that participants disproportionally misjudged their traveled distance with ongoing self-motion. Fitting α and *k* over all participants, we obtained a small leakage rate of α = 0.103 (*SD* = 0.089) and a gain factor slightly above 1 (*k* = 1.117, *SD* = 0.224), again consistent with previous studies of optic flow-based distance estimation (Bossard et al., 2016; Bossard & Mestre, 2018; Clément et al., 2020; Lappe et al., 2007; Lappe et al., 2011; Stangl et al., 2020).

**Figure 3.**
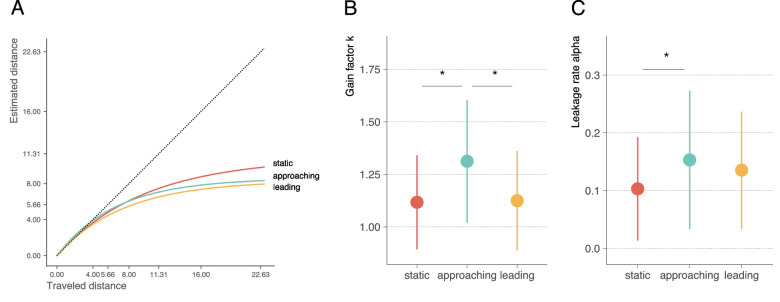
**(A)** Leaky fit per condition. The dotted line indicates veridical performance. **(B, C)** Leaky fit parameter *k* **(B)** and leak rate α **(C)** per condition. Asterisks denote significant differences between conditions.

To analyze differences in travel distance estimation in the different conditions, we fitted each condition to a leaky integrator model and determined the fit parameters gain (*k*) and leak rate (α). A positive value for the leak rate leads to an underestimation of travel distance for long distances even if the gain is perfect. Thus, both parameters can potentially contribute to the distance underestimation and a comparison between the conditions might show some differences in the use of optic flow and biological motion as visual signals. Figure 3 depicts the average values for *k* and α per condition.

The model fits indicated that the gain *k* was larger than 1 in all conditions (static: *M* = 1.18, *SD* = 0.41; approaching: *M* = 1.47, *SD* = 0.80; leading: *M* = 1.17, *SD* = 0.50). This suggests that the transformation from visual motion to travel distance slightly overestimates travel distance. The leakage rate α was larger than zero in all conditions (*M* = 0.12, *SD* = 0.07; approaching: *M* = 0.20, *SD* = 0.14; leading: *M* = 0.14, *SD* = 0.07), indicating that the overall underestimation of travel distance is due to the leak.

To assess whether walker conditions affect the magnitude of model parameters, we calculated ANOVAs separately for *k* and α. The results for the gain parameter confirmed the statistical significance of the main effect walker condition, *F*(2) = 4.68, *p* = 0.014, $\eta_{p}^{2}$ = 0.17, 95% CI, 0.02–1.00. Note the effect size is large. The same pattern emerges for α. The main effect of the condition on α reached significance with a large effect size, *F*(2) = 5.88, *p* = 0.005, $\eta_{p}^{2}$ = 0.20, 95% CI, 0.04–1.00. In other words, the translation from physical to perceived distance (*k*) and the decay (α) leading to underestimation depends on the walker condition. Post hoc analyses showed that the gain *k* was largest in the approaching condition and significantly different from the static (*p* = 0.034, *M_diff_* = 0.287) and leading (*p* = 0.025, *M_diff_* = 0.299) conditions. Leading and static conditions did not show a statistically significant difference (*p* = 0.993, *M_diff_* = 0.013). The large value for *k* in the approaching condition might indicate that in this condition the excessive optic flow from the walker is not completely compensated by the biological motion analysis. The leak rate α was significantly higher in the approaching compared to the static condition (*p* = 0.005, *M_diff_* = 0.080). Neither the differences between approaching and leading (*p* = 0.059, *M_diff_* = 0.056) nor leading and static crowds (*p* = 0.590, *M_diff_* = 0.023) reached significance. The combination of a large *k* with a simultaneously increased α produces underestimation in the approaching condition even though the gain in this condition is the largest.

## Discussion

Our study investigated travel distance estimation for self-motion through a crowd of point-light walkers. To assess the impact of biological motion and optic flow as information sources of self-motion, we designed three conditions. The static condition presented solely standing walkers, thus, solely conveying optic flow. The leading crowd condition depicted a crowd that participants visually followed. Due to the similar to identical motion speed of the observer and the walkers, the optic flow was reduced to a minimum. Here, participants must infer visual self-motion from the articulation of the walkers in the crowd (Seno & Sato, 2012; Masselink & Lappe, 2015; Riddell & Lappe, 2017; Hülemeier & Lappe, 2020). In the approaching condition, participants visually traversed a crowd that walked toward them. This scene combined optic flow from the observer's movement with optic flow created by the approaching walkers, and participants had to use the articulation of the biological motion to estimate the speed of the crowd and remove its optic flow from the full optic flow to gauge their self-motion speed for distance estimation.

In line with previous studies (Frenz & Lappe, 2005; Lappe et al., 2005; Lappe et al., 2007; Lappe & Frenz, 2009; Bossard et al., 2016), our participants underestimated their traveled distances across conditions. The underestimation occurred disproportionally large for longer distances. The data were fitted by the leaky integration model (Lappe et al., 2007). This model describes a disproportional underestimation of long distances by two parameters: gain factor *k*, representing the congruency between physical and perceived distance, and leak rate α, modeling the disproportional misjudgments of long distances. The fitted parameters in the static condition, providing solely optic flow information and no biological motion, were consistent with those from the literature on travel distance estimation from option flow (Lappe et al., 2007; Lappe et al., 2011; Bossard et al., 2016; Bossard & Mestre, 2018; Clément et al., 2020; Stangl et al., 2020).

The leading crowd condition investigated whether observers were able to infer travel distance from biological motion. Considering that there is little to no optic flow in the leading condition, distance estimates of similar magnitude as in the static condition ascertain that observers can indeed infer their velocity from pure biological motion. Participants must have derived the appropriate translational speed of the crowd from their biological motion. To do this, they had to calculate the theoretically appropriate translational speed of the walker from the walker's arm and leg movement (Masselink & Lappe, 2015; Thurman & Lu, 2016). Equating one's speed with the speed of the crowd would then enable travel distance perception similar to that of optic flow because distance estimation from the optic flow is also based on perceived ego speed (Frenz et al., 2003; Frenz & Lappe, 2005). The analysis of the leaky fit parameters confirms that this conversion of the biological movement of others into ego-speed works similarly to that for optic flow. Gain factor *k* and leak rate α of the leading crowd condition are not different from those of the static condition.

The approaching crowd condition contains twice the optic flow as the static condition because not only does the observer walk through the crowd, but the crowd also approaches the observer. The finding that travel distance estimation is similar to that of the static condition suggests that observers were able to use information from the articulation of the biological motion to infer their correct ego-speed. If they can estimate the speed of the crowd from the articulation of the walkers, this speed might be deducted from the forward speed of the optic flow to produce ego-speed. In parallel, the visual system processes the optic flow of the entire scene. From the optic flow, the visual system must subtract the deduced translation speed. Our results suggest an interesting link to flow parsing. Flow parsing (Rushton & Warren, 2005; Warren & Rushton, 2009) refers to the process by which the visual system extracts the independent motion of objects within a scene during self-motion. This decomposition is achieved by subtracting the motion due to self-motion from the complete retinal motion field. As a result, the visual system can parse the optic flow input and estimate the independent motion of objects. Flow parsing on its own (i.e., only using visual information) underestimates ego-speed and, thus, misestimates object speed (Xie, Niehorster, Lappe, & Li, 2020). But, during real walking, when vestibular and somatosensory cues are also available, flow passing is close to perfect (Xie et al., 2020). In addition, flow parsing, can also take limb articulation or facing direction of biological motion into account to estimate the direction and speed of walkers embedded in optic flow (Mayer, Riddell, & Lappe, 2021). This is similar to the situation in our present experimental conditions, in which participants have to use articulation information to extract their own speed from the optic flow by decomposing the flow into own and crowd components. The difference between our study and typical flow parsing studies is that our task is to estimate ego-speed rather than speed of the object. Participants would first subtract the walker motion to then recover the self-motion. In either case, participants use their prior knowledge of biological motion to deduce self-motion estimates.

With perfect calculations, gain factor *k* and leak rate α should be the same as in the static and leading conditions. However, the leaky integration fits of the data produced a gain factor *k* significantly larger than in the other conditions. A larger gain means that the perceived distance of an elementary movement is larger than its physical distance. This could occur, for example, if the speed of the crowd is underestimated and the ego-speed thus overestimated. Estimation of ego-speed is indeed not perfect, neither during walking nor from static viewing of optic flow stimuli (Durgin, Gigone, & Scott, 2005; Durgin, 2009; Tcheang, Gilson, & Glennerster, 2005).

With a larger value for *k*, one might expect participants to overestimate travel distance; however, the increase in *k* was countered by an increase in leak rate α. Thus, although the gain for each elementary movement was large the overall distance estimate was not increased due to the larger leak. Significant effects for α represent the difficulty of estimating distance from approaching walkers alone. This difficulty might derive from the fact that approaching walkers keep disappearing from the field of view. To re-estimate self-motion, participants need to pick another walker as an anchor. However, this walker may have a different translational velocity and will fall out as an anchor again after some time. Distance estimation is more error-prone than approaching static objects or leading crowds.

To conclude, we found that humans are capable of estimating their traveled distance from biological motion alone or in combination with optic flow. In either case, the distance estimates follow a leaky path integration in which visual motion is transformed into ego-speed and then integrated throughout travel in a leaky manner.

## Supplementary Material

Supplement 1

## Supplementary material

**Supplementary Movie S1.**  Stimulus presentation and distance estimation. Self-motion velocity is equal to the average crowd speed across trials. The first trial depicts the static condition, the second one the approaching, and the third one the leading condition. Every trial is followed by traveled distance report.

## References

[bib1] Bossard, M., Goulon, C., & Mestre, D. R. (2016). Viewpoint oscillation improves the perception of distance travelled based on optic flow. *Journal of Vision,* 16(15):4, 1–14, 10.1167/16.15.4. 27919100

[bib2] Bossard, M., & Mestre, D. R. (2018). The relative contributions of various viewpoint oscillation frequencies to the perception of distance traveled. *Journal of Vision,* 18(2):3, 1–18, 10.1167/18.2.3. 29392278

[bib3] Bremmer, F., & Lappe, M. (1999). The use of optical velocities for distance discrimination and reproduction during visually simulated self-motion. *Experimental Brain Research**,* 127(1), 33–42, 10.1007/s002210050771. 10424412

[bib4] Cheng, Z., & Gu, Y. (2018). Vestibular system and self-motion. *Frontiers in Cellular Neuroscience,* 12, 456, 10.3389/fncel.2018.00456. 30524247 PMC6262063

[bib5] Clément, G., Bukley, A., Loureiro, N., Lindblad, L., Sousa, D., & Zandvilet, A. (2020). Horizontal and vertical distance perception in altered gravity. *Scientific Reports,* 10(1), 1–11, 10.1038/s41598-020-62405-0. 32214172 PMC7096486

[bib6] De Lussanet, M. H., Fadiga, L., Michels, L., Seitz, R. J., Kleiser, R., & Lappe, M. (2008). Interaction of visual hemifield and body view in biological motion perception. *European Journal of Neuroscience,* 27(2), 514–522, 10.1111/j.1460-9568.2007.06009.x. 18215244

[bib7] Durgin, F. H. (2009). When walking makes perception better. *Current Directions in Psychological Science,* 18(1), 43–47, 10.1111/j.1467-8721.2009.01603.x.

[bib8] Durgin, F. H., Gigone, K., & Scott, R. (2005). Perception of visual speed while moving. *Journal of Experimental Psychology: Human Perception and Performance**,* 31(2), 339–353, 10.1037/0096-1523.31.2.339. 15826235

[bib9] Ellmore, T. M., & McNaughton, B. L. (2004). Human path integration by optic flow. *Spatial Cognition and Computation**,* 4(3), 255–272, 10.1207/s15427633scc0403_3.

[bib10] Field, A (2013) *Discovering statistics using IBM SPSS Statistics* (4th ed.). London: Sage.

[bib11] Frenz, H., Bremmer, F., & Lappe, M. (2003). Discrimination of travel distances from ‘situated’ optic flow. *Vision Research**,* 43(20), 2173–2183, 10.1016/S0042-6989(03)00337-7. 12855252

[bib12] Frenz, H., & Lappe, M. (2005). Absolute travel distance from optic flow. *Vision Research**,* 45(13), 1679–1692, 10.1016/j.visres.2004.12.019. 15792843

[bib13] Frenz, H., & Lappe, M. (2006). Visual distance estimation in static compared to moving virtual scenes. *The Spanish Journal of Psychology**,* 9(2), 321–331, 10.1017/S1138741600006223. 17120711

[bib14] Frenz, H., Lappe, M., Kolesnik, M., & Bührmann, T. (2007). Estimation of travel distance from visual motion in virtual environments. *ACM Transactions on Applied Perception**,* 4(1), 3–es, 10.1145/1227134.1227137.

[bib15] Frissen, I., Campos, J. L., Souman, J. L., & Ernst, M. O. (2011). Integration of vestibular and proprioceptive signals for spatial updating. *Experimental Brain Research,* 212(2), 163–176, 10.1007/s00221-011-2717-9. 21590262

[bib16] Fujimoto, K., & Sato, T. (2006). Backscroll illusion: Apparent motion in the background of locomotive objects. *Vision Research**,* 46(1-2), 14–25, 10.1016/j.visres.2005.09.027. 16289275

[bib17] Gibson, J. J. (1950). *The perception of the visual world*. Cambridge, UK: Riverside Press.

[bib18] Giese, M. A., & Lappe, M. (2002). Measurement of generalization fields for the recognition of biological motion. *Vision Research**,* 42(15), 1847–1858, 10.1016/S0042-6989(02)00093-7. 12128015

[bib19] Harris, L. R., Herpers, R., Jenkin, M., Allison, R. S., Jenkin, H., Kapralos, B., & Felsner, S. (2012). The relative contributions of radial and laminar optic flow to the perception of linear self-motion. *Journal of Vision**,* 12(10):7, 1–10, 10.1167/12.10.7. 22976397

[bib20] Harris, L. R., Jenkin, M., & Zikovitz, D. C. (2000). Visual and non-visual cues in the perception of linear self motion. *Experimental Brain Research**,* 135(1), 12–21, 10.1007/s002210000504. 11104123

[bib22] Hülemeier, A.-G., & Lappe, M. (2020). Combining biological motion perception with optic flow analysis for self-motion in crowds. *Journal of Vision**,* 20(9):7, 1–15, 10.1167/jov.20.9.7. PMC7488621 32902593

[bib23] Israël, I., & Berthoz, A. (1989). Contribution of the otoliths to the calculation of linear displacement. *Journal of Neurophysiology**,* 62(1), 247–263, 10.1152/jn.1989.62.1.247. 2754476

[bib24] Jaeger, T. F. (2008). Categorical data analysis: Away from ANOVAs (transformation or not) and towards logit mixed models. *Journal of Memory and Language**,* 59(4), 434–446, 10.1016/j.jml.2007.11.007. 19884961 PMC2613284

[bib25] Johansson, G. (1973). Visual perception of biological motion and a model for its analysis. *Perception & Psychophysics**,* 14(2), 201–211, 10.3758/BF03212378.

[bib26] Kearns, M. J., Warren, W. H., Duchon, A. P., & Tarr, M. J. (2002). Path integration from optic flow and body senses in a homing task. *Perception**,* 31(3), 349–374, 10.1068/p3311. 11954696

[bib27] Kleiner, M., Brainard, D., & Pelli, D. (2007). What's new in Psychtoolbox-3? *Perception**,* 36, 1–16, 10.1068/v070821.

[bib28] Koerfer, K., & Lappe, M. (2020). Pitting optic flow, object motion, and biological motion against each other. *Journal of Vision**,* 20(8):18, 1–13, 10.1167/jov.20.8.18. PMC7438637 32805041

[bib29] Kristensen, M., & Hansen, T. (2004). Statistical analyses of repeated measures in physiological research: A tutorial. *Advances in Physiology Education**,* 28(1), 2–14, 10.1152/advan.00042.2003. 14973047

[bib30] Lappe, M., & Frenz, H. (2009). Visual estimation of travel distance during walking. *Experimental Brain Research**,* 199(3–4), 369, 10.1007/s00221-009-1890-6. 19533107

[bib31] Lappe, M., Frenz, H., Bührmann, T., & Kolesnik, M. (2005). Virtual odometry from visual flow. *Proceedings of SPIE**,* 5666, 493–502, 10.1117/12.610863.

[bib32] Lappe, M., Jenkin, M., & Harris, L. R. (2007). Travel distance estimation from visual motion by leaky path integration. *Experimental Brain Research**,* 180(1), 35–48, 10.1007/s00221-006-0835-6. 17221221

[bib33] Lappe, M., Stiels, M., Frenz, H., & Loomis, J. M. (2011). Keeping track of the distance from home by leaky integration along veering paths. *Experimental Brain Research**,* 212(1), 81–89, 10.1007/s00221-011-2696-x. 21533833

[bib34] Lee, D. N. (1974). Visual information during locomotion. In R. B. MacLeod & H. L. Pick (Eds.), *Perception: Essays in honor of James J. Gibson* (pp. 250–267)*.* Ithaca, NY: Cornell University Press.

[bib35] Lee, D. N. (1976). A theory of visual control of braking based on information about time-to-collision. *Perception**,* 5(4), 437–459, 10.1068/p050437. 1005020

[bib36] Lee, D. N. (1980). The optic flow field: The foundation of vision. *Philosophical Transactions of the Royal Society of London B: Biological Sciences**,* 290(1038), 169–179, 10.1098/rstb.1980.0089. 6106236

[bib37] Masselink, J., & Lappe, M. (2015). Translation and articulation in biological motion perception. *Journal of Vision**,* 15(11):10, 1–14, 10.1167/15.11.10. 26270192

[bib38] Maurer, R., & Séguinot, V. (1995). What is modelling for? A critical review of the models of path integration. *Journal of Theoretical Biology**,* 175(4), 457–475, 10.1006/jtbi.1995.0154.

[bib39] Mayer, K. M., Riddell, H., & Lappe, M. (2021). Flow parsing and biological motion. *Attention, Perception, & Psychophysics**,* 83, 1752–1765, 10.3758/s13414-020-02217-6. PMC8084786 33629261

[bib40] Mittelstaedt, H., & Mittelstaedt, M. L. (1973). Mechanismen der Orientierung ohne richtende Außenreize. *Fortschritte der Zoologie**,* 21(2/3), 46–58.

[bib41] Mittelstaedt, M. L., & Mittelstaedt, H. (2001). Idiothetic navigation in humans: Estimation of path length. *Experimental Brain Research**,* 139(3), 318–332, 10.1007/s002210100735. 11545471

[bib42] Mossio, M., Vidal, M., & Berthoz, A. (2008). Traveled distances: New insights into the role of optic flow. *Vision Research**,* 48(2), 289–303, 10.1016/j.visres.2007.11.015. 18177912

[bib43] Nico, D., Israël, I., & Berthoz, A. (2002). Interaction of visual and idiothetic information in a path completion task. *Experimental Brain Research**,* 146(3), 379–382, 10.1007/s00221-002-1184-8. 12232694

[bib44] Redlick, F. P., Jenkin, M., & Harris, L. R. (2001). Humans can use optic flow to estimate distance of travel. *Vision Research**,* 41(2), 213–219, 10.1016/S0042-6989(00)00243-1. 11163855

[bib45] Riddell, H., & Lappe, M. (2017). Biological motion cues aid identification of self-motion from optic flow but not heading detection. *Journal of Vision**,* 17(12):19, 1–17, 10.1167/17.12.19. 29090314

[bib46] Riddell, H., & Lappe, M. (2018). Heading through a crowd. *Psychological Science**,* 29(9), 1504–1514, 10.1177/0956797618778498. 30004826

[bib47] Riddell, H., Li, L., & Lappe, M. (2019). Heading perception from optic flow in the presence of biological motion. *Journal of Vision**,* 19(14):25, 1–14, 10.1167/19.14.25. 31868898

[bib48] Rushton, S. K., & Warren, P. A. (2005). Moving observers, relative retinal motion and the detection of object movement. *Current Biology**,* 15(14), R542–R543, 10.1016/j.cub.2009.07.057. 16051158

[bib49] Seno, T., & Sato, T. (2012). Vection can be induced without explicit motion signal using backscroll illusion. *Japanese Psychological Research**,* 54(2), 218–222, 10.1111/j.1468-5884.2011.00498.x.

[bib50] Stangl, M., Kanitschneider, I., Riemer, M., Fiete, I., & Wolbert, T. (2020). Sources of path integration error in young and aging humans. *Nature Communications**,* 11(2626), 1–15, 10.1038/s41467-020-15805-9. PMC7250899 32457293

[bib51] Steinicke, F., Bruder, G., Hinrichs, K., Lappe, M., Ries, B., & Interrante, V. (2009). Transitional environments enhance distance perception in immersive virtual reality systems. In: *Proceedings of the 6th Symposium on Applied Perception in Graphics and Visualization* (pp. 19–26). New York: Association for Computing Machinery.

[bib52] Thomson, J. A. (1980). How do we use visual information to control locomotion? *Trends in Neurosciences**,* 3(10), 247–250, 10.1016/S0166-2236(80)80076-2.

[bib53] Thurman, S. M., & Lu, H. (2016). Revisiting the importance of common body motion in human action perception. *Attention, Perception, & Psychophysics**,* 78(1), 30–36, 10.3758/s13414-015-1031-1. 26603043

[bib54] Tcheang, L., Gilson, S. J., & Glennerster, A. (2005) Systematic distortions of perceptual stability investigated using immersive virtual reality. *Vision Research**,* 45, 2177–2189, 10.1016/j.visres.2005.02.006. 15845248 PMC2833395

[bib55] Warren, P. A., & Rushton, S. K. (2009). Optic flow processing for the assessment of object movement during ego movement. *Current Biology**,* 19(18), 1555–1560, 10.1016/j.cub.2009.07.057. 19699091

[bib56] Xie, M., Niehorster, D.C., Lappe, M. & Li, L. (2020) Roles of visual and non-visual information in the perception of scene-relative object motion during walking. *Journal of Vision**,* 20(10):15, 1–11, 10.1167/jov.20.10.15. PMC7571284 33052410

